# PSPC1 Inhibition Synergizes with Poly(ADP-ribose) Polymerase Inhibitors in a Preclinical Model of BRCA-Mutated Breast/Ovarian Cancer

**DOI:** 10.3390/ijms242317086

**Published:** 2023-12-03

**Authors:** Mithun Ghosh, Min Sil Kang, Nar Bahadur Katuwal, Sa Deok Hong, Yeong Gyu Jeong, Seong Min Park, Seul-Gi Kim, Yong Wha Moon

**Affiliations:** 1 Department of Biomedical Science, The Graduate School, CHA University, Seongnam-si 13488, Republic of Korea; 2 Hematology and Oncology, Department of Internal Medicine, CHA Bundang Medical Center, CHA University, Seongnam-si 13496, Republic of Korea

**Keywords:** PARP inhibitor, *PSPC1*, DNA double-strand break, sensitivity

## Abstract

Poly (ADP-ribose) polymerase (PARP) inhibitors are effective against *BRCA1/2*-mutated cancers through synthetic lethality. Unfortunately, most cases ultimately develop acquired resistance. Therefore, enhancing PARP inhibitor sensitivity and preventing resistance in those cells are an unmet clinical need. Here, we investigated the ability of paraspeckle component 1 (*PSPC1*), as an additional synthetic lethal partner with *BRCA1/2*, to enhance olaparib sensitivity in preclinical models of *BRCA1/2*-mutated breast and ovarian cancers. In vitro, the combined olaparib and *PSPC1* small interfering RNA (siRNA) exhibited synergistic anti-proliferative activity in *BRCA1/2*-mutated breast and ovarian cancer cells. The combination therapy also demonstrated synergistic tumor inhibition in a xenograft mouse model. Mechanistically, olaparib monotherapy increased the expressions of p-ATM and DNA-PKcs, suggesting the activation of a DNA repair pathway, whereas combining *PSPC1* siRNA with olaparib decreased the expressions of p-ATM and DNA-PKcs again. As such, the combination increased the formation of γH2AX foci, indicating stronger DNA double-strand breaks. Subsequently, these DNA-damaged cells escaped G2/M checkpoint activation, as indicated by the suppression of p-cdc25C (Ser216) and p-cdc2 (Tyr15) after combination treatment. Finally, these cells entered mitosis, which induced increased apoptosis. Thus, this proves that *PSPC1* inhibition enhances olaparib sensitivity by targeting DNA damage response in our preclinical model. The combination of olaparib and *PSPC1* inhibition merits further clinical investigation to enhance PARP inhibitor efficacy.

## 1. Introduction

*BRCA1* and *BRCA2* (*BRCA1/2*) are important genes involved in homologous recombination (HR) repair, particularly in the repair of DNA double-strand breaks (DSBs) [1]. Hence, BRCA1/2, which act as tumor suppressors, suppress genetic instability [2]. From the therapeutic perspective, cancer cells with *BRCA1/2* mutations are very sensitive to poly (ADP-ribose) polymerase (PARP) inhibitors because PARP inhibitors cause an increase in DNA single-strand breaks, which are then converted to irreparable toxic DNA DSBs in those cells during replication [3]. The prevalence of *BRCA1/2* mutations is highest in ovarian cancer (21%) [4] and around 5% in several other cancers, including breast [5], prostate [6], pancreatic [7], non-small cell lung [8], bladder [9], and bile duct [10] cancers. In particular, subsets of certain cancers, such as triple-negative breast cancer (10–15%) [5] and castration-resistant prostate cancer (13.6%) [6], have a higher prevalence of BRCA1/2 mutations than does the whole population of that cancer.

In *BRCA1/2*-mutated or HR-deficient (HRD) ovarian cancer, olaparib or niraparib maintenance after first-line paclitaxel/carboplatin significantly prolonged progression-free survival (PFS) in the SOLO1 (13.8 vs. 56 months in the placebo and olaparib groups, respectively) [11] or PRIMA trial (10.4 vs. 21.9 months in the placebo and niraparib groups, respectively) [12]. Improvements in PFS translated to overall survival (OS) benefits in the SOLO1 trial [13]; however, OS data for the PRIMA trial has not been reported yet. In addition, PARP inhibitors, such as talazoparib [14], rucaparib [15], niraparib [16], and olaparib [17] have been approved in for the treatment of the aforementioned cancers with *BRCA1/2* mutation by the United States Food and Drug Administration.

Though PARP inhibitors are effective therapy for *BRCA1/2*-mutated cancers, not all patients respond, and most patients ultimately progress on PARP inhibitor therapy. Therefore, enhancing PARP inhibitor sensitivity or preventing BRCA-mutated cells from developing resistance is an unmet need. Considering this fact, the validation of an additional synthetic lethal partner with *BRCA1/2* genes or the finding of drug synergism with PARP inhibitors could be a promising therapeutic approach. Initial combinations evaluated DNA-damaging chemotherapy in combination with PARP inhibitors, however, overlapping toxicity such as myelosuppression, limited further development of this combination [18]. To enhance PARP inhibitor activity, then next approaches included targeting DNA damage response (DDR)-associated genes such as ataxia telangiectasia and Rad3-related serine/threonine kinase (*ATR*) [19], checkpoint kinase 1 (Chk1) [20], and wee1 G2 checkpoint kinase (*WEE1*) [21]. A synergistic mechanism of this combination is that DNA-damaged cells damaged by PARP inhibitors undergo mitosis without repairing DNA damage resulting in increased apoptosis. In addition, several strategies other than targeting the DDR pathway, including antiangiogenic agents [22] and immune checkpoint inhibitors [23], have been reported. Of all combination partners to enhance PARP inhibitor activity, bevacizumab, a vascular endothelial growth factor inhibitor, only proved clinical efficacy. That is, the phase III PAOLA-1 clinical trial demonstrated the good efficacy of combined olaparib and bevacizumab as a maintenance therapy after first-line chemotherapy in HRD or BRCA-mutated ovarian cancer [24] and finally led to the regulatory approval of this combination. Despite this, we still need to enhance PARP inhibitor activity in various ways in a PARP inhibitor-sensitive setting. Therefore, we tried to search for an additional synthetic lethal partner with *BRCA1/2* genes.

Paraspeckle component 1 (*PSPC1*) was identified as structural protein found in paraspeckles. Paraspeckles are nuclear bodies located in the interchromatin space of the cell nucleus adjacent to speckles and these nuclear bodies are primarily composed of three proteins, such as *PSPC1*, *SFPQ* (splicing factor proline and glutamine-rich, known as PSF), and *NONO* (non-POU-domain-containing octamer-binding protein, known as p54nrb) [25]. Regarding the function of *PSPC1*, there were only limited studies to date, therefore the functions of *PSPC1* have not been completely understood. However, based on other studies, *PSPC1* has been shown to exhibit RNA-binding activity, involved in mRNA splicing [26], regulating androgen receptor-mediated transcription activity [27]. Moreover, the functions of *PSPC1* include viral gene regulation, adipocyte differentiation, and gene regulation [28]. In addition, recruitment of *PSPC1* could be promoted to the DNA damage sites by PSF following p54nrb knockdown [29]. Interestingly, *PSPC1* was also reported to play a role in cisplatin-induced and methyl methanesulfonate-induced DDR [30,31]. Such reports led us to hypothesize that *PSPC1* could be a participant in DDR. Recent studies reported that *PSPC1* was associated with various cancers, such as liver cancer, nasopharyngeal cancer, lung cancer and breast cancer, which is a poor prognostic factor for patient survival [32]. Therefore, it motivated us to test the synthetic lethality of *PSPC1* inhibition in *BRCA1/2*-mutated cancer. Herein, we investigated the synergism of *PSPC1* inhibition in combination with olaparib in a preclinical model with *PSPC1*-expressing, *BRCA1/2*-mutated breast and ovarian cancer, demonstrating the synergistic anticancer activity of the combination.

## 2. Results

### 2.1. PSPC1 Inhibition Synergizes with Olaparib in BRCA1/2-Mutated PSPC1-Expressing Cells

**Figure 1:**
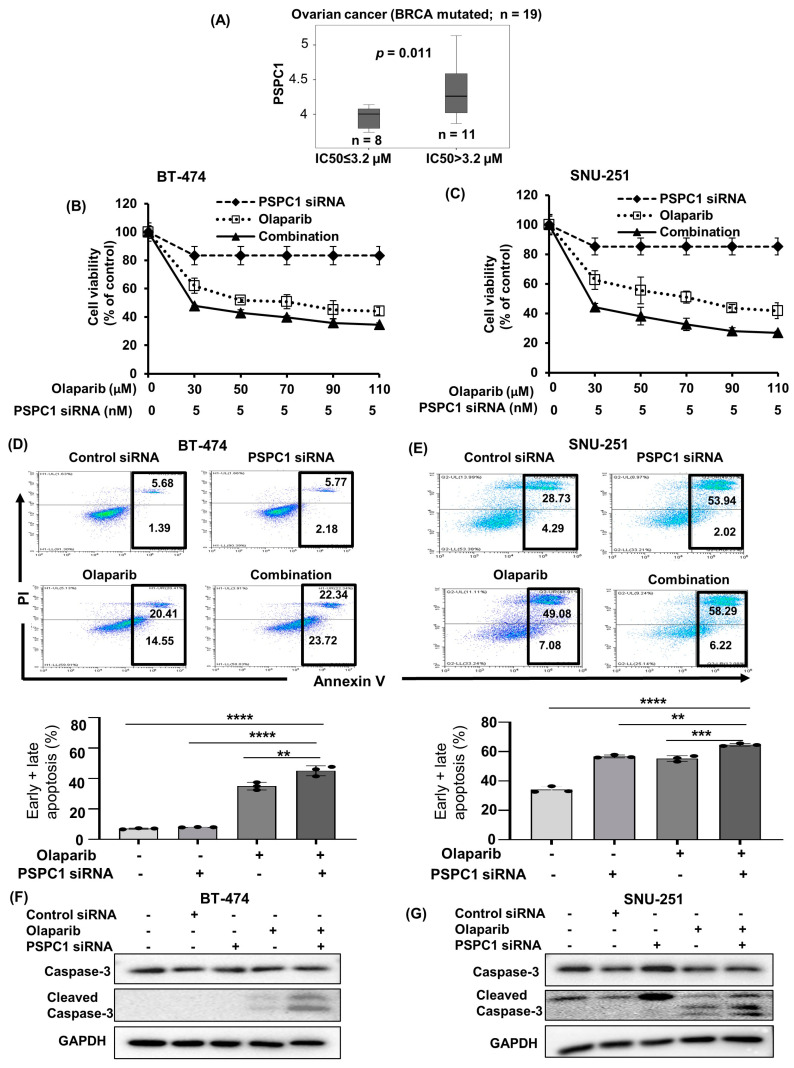
PSPC1 inhibition synergizes with olaparib in *BRCA1/2*-mutated PSPC1-expressing cells. (**A**) Correlation between PSPC1 expression and olaparib sensitivity, which was defined as IC_50_ ≤ 3.2 µM, in BRCA1/2-mutated ovarian cancer cell lines using GDSC data. *p*-values were analyzed by Student’s *t*-test. (**B**,**C**) MTT assay showed that combined PSPC1 siRNA and olaparib enhanced the antiproliferative effects in (**B**) BT−474 and (**C**) SNU−251 cells. Cells were treated with 5 nM of PSPC1 siRNA and indicated concentrations of olaparib and incubated for 72 h. (**D**,**E**) Apoptosis assay carried out by flow cytometry after staining with annexin V-APC/PI, representative scatter plots of PI (*y*-axis) and annexin V (*x*-axis). The number of total apoptotic cells were increased after the treatment with combined PSPC1 siRNA (5 nM) and olaparib (IC_50_ concentration in BT−474 and half IC_50_ concentration in SNU−251) for 72 h in (**D**) BT−474 and (**E**) SNU−251 cells. The data shown here were representative of three independent experiments. The bar graphs depicted the average of total apoptotic cells of three independent experiments. *p*-values were calculated by one-way ANOVA analysis, indicating ** *p* < 0.01, *** *p* < 0.001, **** *p* < 0.0001. Data are presented as mean ± standard deviation from three independent experiments. (**F**,**G**) The expressions of apoptosis markers, caspase−3 and cleaved caspase−3 were analyzed with Western blot in (**F**) BT−474 and (**G**) SNU−251 cells. Cells were treated for 72 h with PSPC1 siRNA (5 nM), olaparib (IC_50_ concentration in BT−474 and half IC_50_ concentration in SNU−251) and their combination. GAPDH was used as a loading control.

To evaluate our hypothesis that targeting *PSPC1* may enhance the anticancer activity of PARP inhibitors in *BRCA*-mutated breast or ovarian cancer, we initially analyzed the relationship between *PSPC1* expression and olaparib sensitivity in 19 *BRCA1/2*-mutated ovarian cancer cell lines out of Genomics of Drug Sensitivity in Cancer (GDSC) data. Accordingly, we found a significant inverse relationship (*p* = 0.011) between olaparib activity and *PSPC1* expression (Figure 1A), that is, cells with higher olaparib activity (IC_50_ < 3.2 µM) showed lower *PSPC1* expression than did those with lower olaparib activity (IC_50_ > 3.2 µM).

Next, we carried out a cell proliferation assay with *PSPC1* siRNA (5 nM) and various concentrations of olaparib in *BRCA*-mutated breast (BT−474, MDA-MB−436) and ovarian cancer (SNU-251, PEO1) cells. The characteristics of these cell lines are summarized in Table S1. Combination treatment synergistically (CI < 1, Figure S1) inhibited the cell proliferation of *BRCA1/2*-mutated BT−474 and SNU−251 cells as compared to the monotherapy (Figure 1B,C). Moreover, the combination treatment exhibited synergistic effects in MDA-MB−436 and PEO1 cells as well (Figure S2A,B). Notably, synergism was observed even at the minimum concentration among the concentrations tested in this study, which might help reduce the toxicity of the therapy among patients.

In addition, an apoptosis assay using annexin V and propidium iodide (PI) also confirmed that the combination of *PSPC1* siRNA and olaparib promoted significantly more total apoptotic cells than did *PSPC1* siRNA or olaparib alone in both BT−474 and SNU−251 cells (Figure 1D,E). Furthermore, Western blotting showed that the expression of cleaved caspase-3 was increased after combination treatment in both cell lines, indicating the activation of apoptosis (Figure 1F,G). Based on these results, we can hypothesize that the enhanced apoptosis induced by combined PSPC1 inhibition and olaparib could be attributed to their synergistic effects.

### 2.2. PSPC1 Inhibition Enhances DNA DSBs by Inhibiting Olaparib-Induced DDR

**Figure 2:**
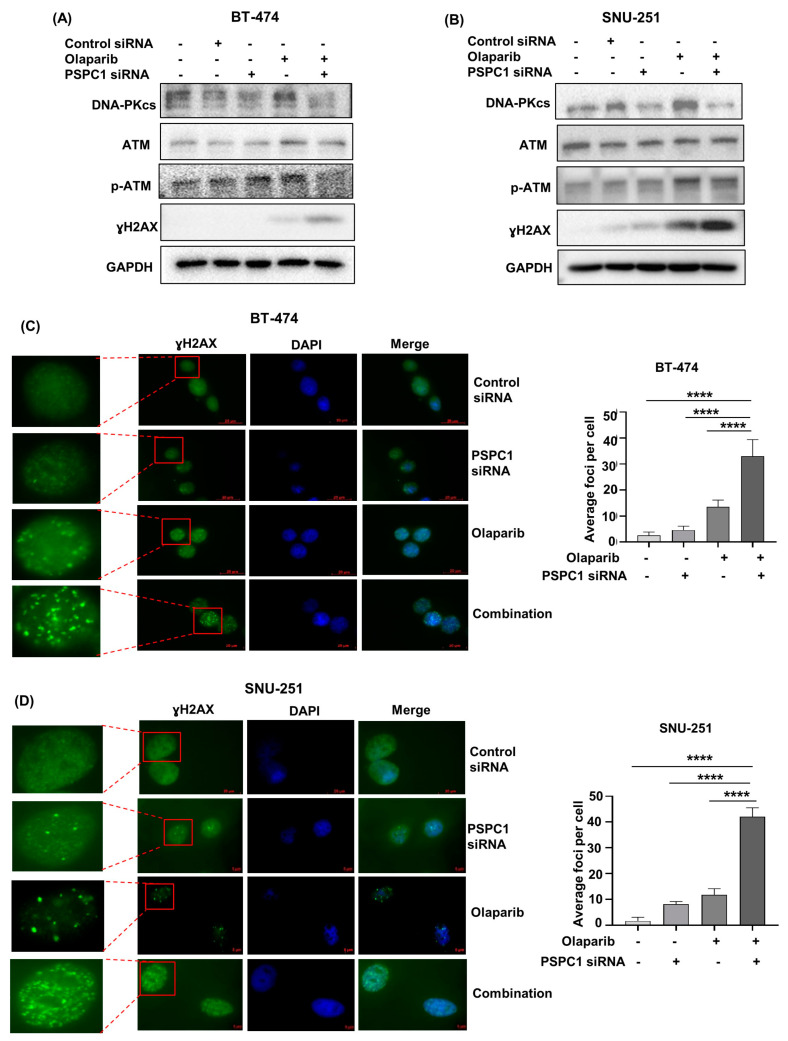
PSPC1 inhibition enhances DNA DSBs by inhibiting olaparib-induced DDR. (**A**,**B**) Western blot was performed to analyze the expressions of DDR-related proteins in (**A**) BT−474 and (**B**) SNU−251 cells. Cells were treated with 5 nM of PSPC1 siRNA, olaparib (IC_50_ concentration in BT−474 and half IC_50_ concentration in SNU−251) and their combination for 72 h. GAPDH was used as a loading control. (**C**,**D**) Immunofluorescence was carried out to capture the ɣH2AX foci formation in (**C**) BT−474 and (**D**) SNU−251 cells as a DNA DSBs marker. The cells were incubated for 48 h after treatment with PSPC1 siRNA (5 nM), olaparib (IC_50_ concentration in BT−474 and half IC_50_ concentration in SNU−251), and their combination. The images were taken at 100x magnification. The images shown here were representative of three independent experiments. The bar graphs depicted the average foci number per cell of three independent experiments. The foci were counted in 10 cells for each group. *P*-values were calculated by one-way ANOVA analysis, indicating **** *p* < 0.0001. Data are presented as mean ± standard deviation from 3 independent experiments.

Next, to investigate the mechanisms behind the aforementioned apoptosis, we assessed the expression of DDR-associated genes because olaparib affects DNA damage based on its mechanism of action. We initially analyzed the expression of ɣH2AX as a DNA DSBs marker and observed that olaparib monotherapy increased the expression of ɣH2AX. However, when *PSPC1* siRNA was combined with olaparib, a robust expression of ɣH2AX was observed in both BT−474 and SNU−251 cells (Figure 2A,B), suggesting the combination therapy was able to induce more profound DNA DSBs. In addition, the immunocytochemistry staining showed that combination treatment promoted greater formation of ɣH2AX foci than did monotherapy in both cell lines (Figure 2C,D).

*BRCA1/2*-mutated cells are known to be HRD and based on the reports, those HRD cells rely on the non-homologous end joining (NHEJ) pathway for DSB repair [33]. Hence, we evaluated the expression of NHEJ pathway-related genes and noticed that olaparib treatment induced the expression of the DNA-dependent protein kinase catalytic subunit (DNA-PKcs), indicating that the NHEJ-mediated DSB repair pathway was activated [34]. However, the combination therapy inhibited the expression of DNA-PKcs again, suggesting that the olaparib-induced DDR via NHEJ was decreased when *PSPC1* was inhibited, leading to more profound DNA damage (Figure 2A,B). Based on previous reports that ataxia-telangiectasia mutated (*ATM*) plays some crucial role in the repair of a subset of DSBs via NHEJ [35], the expressions of *ATM* and phospho-ATM (p-ATM) were also analyzed. Notably, our data showed that olaparib treatment increased the expression of p-ATM, which indicated the activation of *ATM* upon DNA damage. In contrast, combining *PSPC1* siRNA with olaparib promoted a considerable decrease in the expression of p-ATM (Figure 2A,B). Overall, *PSPC1* inhibition enhanced DNA damage in the presence of olaparib by suppressing the expression of DDR proteins induced by olaparib.

### 2.3. Mitotic Catastrophe Caused by Combination Treatment Could Explain the Synergistic Mechanisms

**Figure 3:**
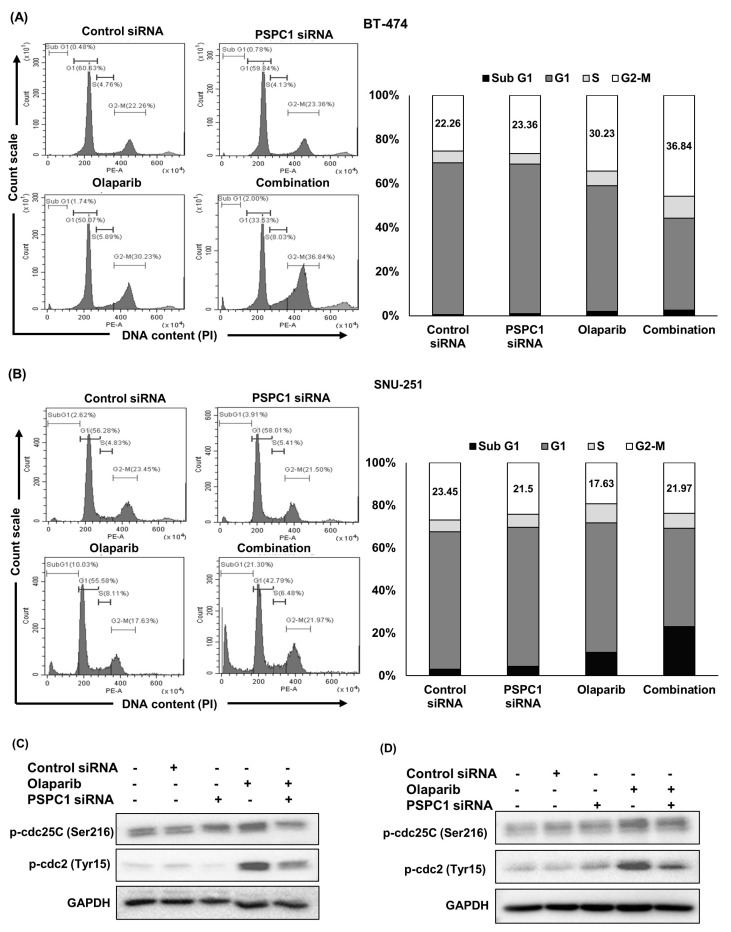

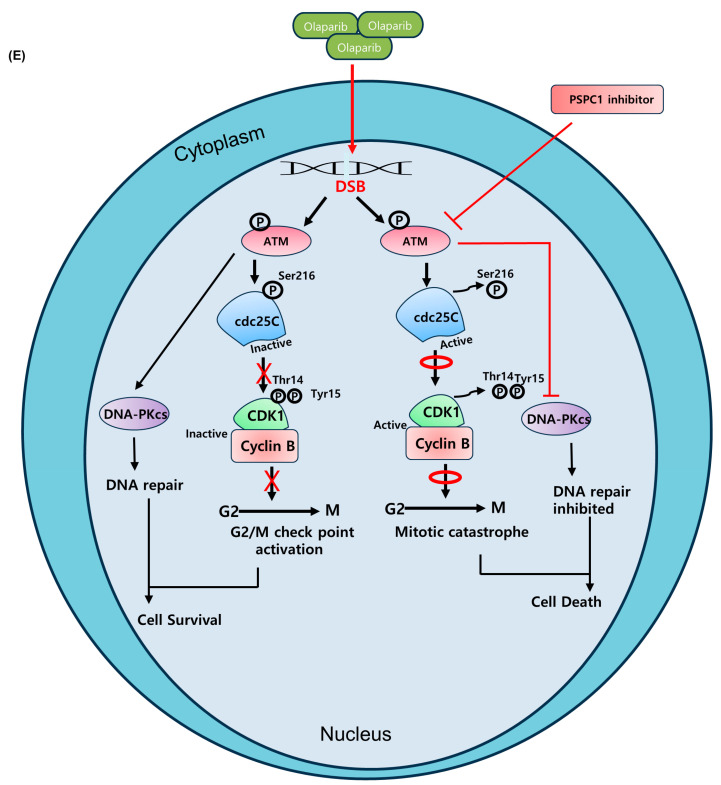
Mitotic catastrophe caused by combination treatment could explain the synergistic mechanisms. (**A**,**B**) BT−474 and SNU−251 cells were treated with 5 nM of PSPC1 siRNA, olaparib (IC_50_ concentration in BT−474 and half IC_50_ concentration in SNU−251), and their combination for 72 h. Cell cycle distribution was assessed by flow cytometry after staining with PI. The histogram exhibited the distribution of cells in various phases (G0/G1, S, and G2/M) and the bar graphs represented the percentage of cells in G0/G1, S, and G2/M phases of cell cycle. (**C**,**D**) Western blot was carried out to check the expression of cell cycle progression-related proteins such as p-cdc25C and p-cdc2. (**C**) BT−474 and (**D**) SNU−251 cells were treated with 5 nM of PSPC1 siRNA, olaparib (IC_50_ concentration in BT-474 and half IC_50_ concentration in SNU−251), and their combination for 72 h. GAPDH was used as a loading control. (**E**) Graphical view of the proposed mechanism. Olaparib induced inhibitory phosphorylation of CDK1 causes mitotic exit which is reversed by PSPC1 inhibition (due to PSPC1 inhibition induced ATM and DNA-PKcs suppression), eventually premature mitotic entry occurred of the DNA-damaged cells, leading to mitotic catastrophe.

Our observation of an increase in DNA DSBs after combination treatment motivated us to investigate how cell cycle distribution was influenced by olaparib and *PSPC1* siRNA treatment. However, our cell cycle analysis in BT−474 cells showed that combination treatment promoted a greater accumulation (36.84%) of G2/M phase cells than did olaparib (30.23%) and *PSPC1* siRNA (23.36%) alone. In addition, the sub-G1 population, which is an indicator of apoptotic cell death, was slightly increased (2%) after combination treatment with olaparib and *PSPC1* siRNA, compared to olaparib alone (1.74%) (Figure 3A). Cell cycle analysis in SNU−251 cells also showed results similar to those for BT−474 cells. The data showed that 17.63% of the cells were accumulated in the G2/M phase after olaparib treatment, which increased to 21.97% when olaparib was combined with *PSPC1* siRNA. Similarly, combination therapy promoted greater accumulation of cells in the sub-G1 phase (21.30%) than did olaparib (10.03%) and *PSPC1* siRNA (3.91%) alone, indicating that adding olaparib to *PSPC1* siRNA enhanced apoptotic cell death (Figure 3B). To further evaluate the molecular mechanism by which *PSPC1* inhibition-induced *ATM* suppression regulated the cell cycle, we investigated the cell cycle transition-regulatory proteins, cell division cycle 25C (*cdc25C*) and cdc2, also known as cyclin dependent kinase 1 (*CDK1*) [36], using Western blotting. *ATM* is known to play key roles in checkpoint activation via Chk1/checkpoint kinase 2 (Chk2) upon DNA DSBs [37]. Specifically, phospho-Chk1 (activated by p-ATM) has been reported to inactivate *cdc25C* by phosphorylation on serine-216, which prevents dephosphorylation of *cdc2*, resulting in the suppression of CDK1/cyclin B complex, thereby inducing G2/M checkpoint activation [38]. We observed that the expressions of phospho-cdc25C [p-cdc25C (Ser216)] and phospho-cdc2 [p-cdc2 (Tyr15)] were increased after olaparib treatment, suggesting that the G2/M checkpoint was activated. However, combining *PSPC1* siRNA with olaparib decreased the expressions of those two proteins again, indicating that the G2/M transition occurred due to the p-ATM suppression (by *PSPC1* siRNA)-induced activation of cdc25C (Figure 3C,D).

Taken together, *PSPC1* inhibition in combination with olaparib prevented G2/M checkpoint activation and then enhanced the G2/M transition. Hence, cells with damaged DNA enter mitosis, leading to mitotic catastrophe (Figure 3E).

### 2.4. Combination of PSPC1 siRNA and Olaparib Inhibits Tumor Growth in a PSPC1-Expressing BRCA2-Mutated Breast Cancer Xenograft Model

**Figure 4:**
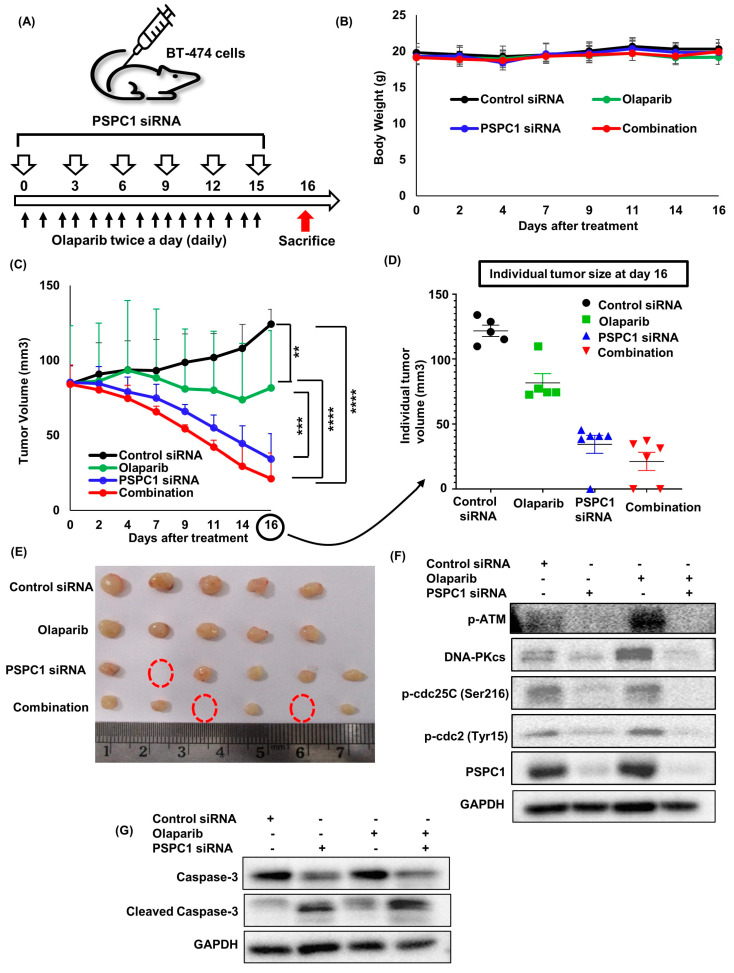
Combination of PSPC1 siRNA and olaparib inhibits tumor growth in a PSPC1-expressing BRCA2-mutated breast cancer xenograft model. (**A**) Graphical view of drug treatment schedule and animal experimental procedure. (**B**) The body weights of mice exhibited that the treated doses were well tolerated as weight loss was not observed (**C**) Mean tumor growth inhibition curves in mice which were treated with olaparib, PSPC1 siRNA, and their combination. Tumor volumes were measured three times a week by a vernier caliper. Indicated *p*-values were calculated by one-way ANOVA analysis on day 16 of treatment, whereas ** *p* < 0.01, *** *p* < 0.001, **** *p* < 0.0001. (**D**) Individual tumor volumes were shown on day 16. Data are presented as the mean ± SEM. (**E**) Photo of the excised tumors of each group shown after sacrificing on day 16. The red dotted circles indicate complete regression of the tumor (**F**) Western blot using BT−474 xenografted tumors after 16 days of treatment. Combination treatment suppressed the expression of DDR-associated genes, such as p-ATM, DNA-PKcs, p-cdc25C, and p-cdc2 compared to monotherapy. (**G**) Western blot using BT−474 xenografted tumors after 16 days of treatment. The expression of the apoptosis marker cleaved caspase-3 was increased after combination treatment compared to monotherapy.

We subsequently evaluated the in vivo efficacy of olaparib, *PSPC1* siRNA, and their combination in a BT−474 xenograft mouse model. The schedule of the animal experiments is depicted in Figure 4A. First of all, *PSPC1* siRNA or the combination used in this study were well tolerated, and no generalized toxicity was observed given that the body weight remained normal (Figure 4B). Combining *PSPC1* siRNA with olaparib promoted greater tumor regression than did *PSPC1* siRNA alone (*p* = 0.211), olaparib alone (*p* < 0.0001), and control siRNA (*p* < 0.0001) (Figure 4C). The individual tumor volumes were shown in Figure 4D. Remarkably, combination treatment exhibited complete tumor regression in two out of the six mice (Figure 4E). *PSPC1* siRNA alone also significantly inhibited the tumor growth, including complete tumor regression in one mouse, compared to control siRNA (*p* < 0.0001). Finally, the mice were sacrificed on day 16, and the tumors were excised for further experiments. After subjecting tumor tissue lysates to Western blot analysis, we observed a similar pattern of expression in the genes tested in vitro. Briefly, combination therapy markedly reduced the expression of DDR-related genes, such as p-ATM and DNA-PKcs. In addition, combination therapy promoted greater inhibition of the expression of cell cycle-related genes, p-cdc25C (Ser216) and p-cdc2 (Tyr15), than did monotherapy, indicating that the G2/M transition occurred (Figure 4F). Lastly, the expression of cleaved caspase-3 was noticeably elevated, suggesting increased apoptosis (Figure 4G).

### 2.5. High PSPC1 Expression Is Associated with Poor Prognosis in Breast and Ovarian Cancer

**Figure 5:**
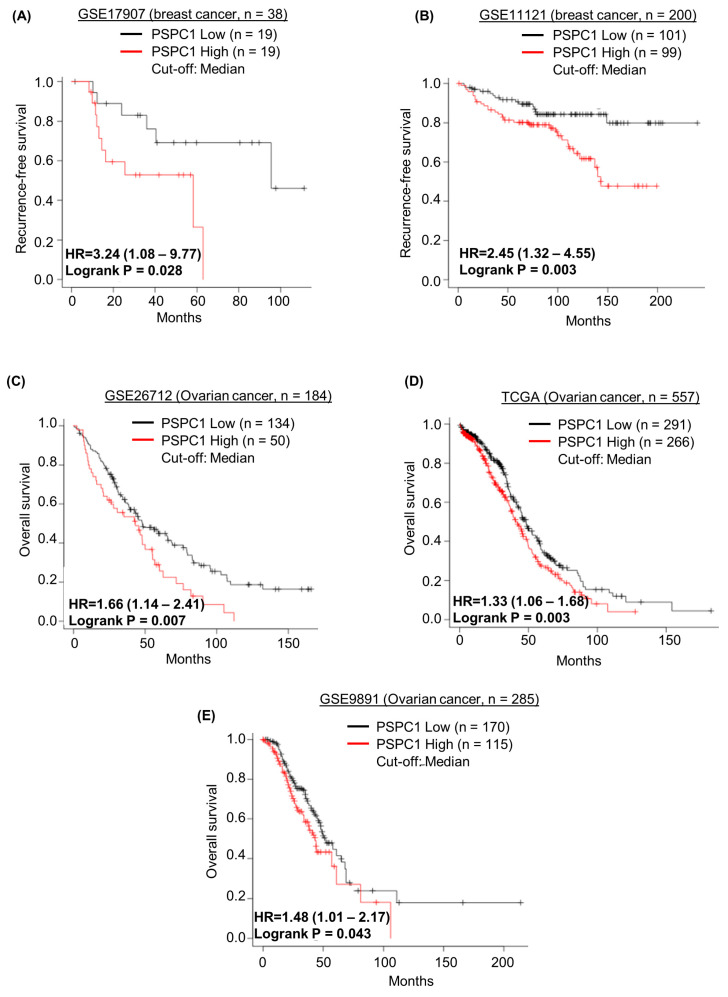
High PSPC1 expression is associated with poor prognosis in breast and ovarian cancer. (**A**,**B**) Kaplan–Meier survival curve of RFS in breast cancer according to relative PSPC1 mRNA expression; the data were analyzed using online platform Kaplan–Meier plotter: https://kmplot.com/analysis/ (accessed on 01 August 2023). (**C**–**E**) Kaplan–Meier survival curve of OS in ovarian cancer according to relative *PSPC1* mRNA expression; the data were analyzed using online platform Kaplan–Meier plotter.

We investigated the impact of PSPC1 overexpression on prognosis using independent public mRNA expression data sets. The *PSPC1*-High group (≥median) exhibited a higher recurrence risk than that of the *PSPC1*-Low group (<median) in two data sets of patients with breast cancer (Figure 5A,B). Similarly, the PSPC1-High group showed shorter OS than *PSPC1*-Low group in the three data sets of ovarian cancer patients (Figure 5C–E). Taken together, our findings based on the public mRNA database showed that high *PSPC1* expression was associated with poor prognosis in breast and ovarian cancer.

## 3. Discussion

Considering the acquired PARP inhibitor resistance in clinics, enhancing PARP inhibitor sensitivity or preventing cells from developing resistance is an urgent and unmet medical need. In the current study, we identified that *PSPC1* could be used as an additional synthetic lethal partner with *BRCA1/2*. We demonstrated that *PSPC1* inhibition enhanced olaparib efficacy in a preclinical model of breast or ovarian cancer with *BRCA1/2* mutations compared to olaparib alone, suggesting a promising therapeutic alternative to enhancing PARP inhibitor sensitivity in non-resistance settings.

*PSPC1* is an RNA-binding protein belonging to the drosophila behavior/human splicing (DBHS) family, which consists of two tandem RNA recognition motifs (RRMS), a nonA/paraspeckle domain (NOPS) and a C-terminal coiled-coil domain [25]. According to various reports, *PSPC1* participates in the regulation of transcription, RNA processing, RNA transport, and DNA repair [39]. Although very limited information is available regarding the drug resistance-promoting activity of *PSPC1*, Kessler et al. reported that *PSPC1* was associated with chemoresistance in nuclear paraspeckle assembly transcript 1-induced hepatocellular carcinoma [40], indicating that *PSPC1* may play some role in the development of drug resistance. Mechanistically, *PSPC1* inhibition-induced mitotic catastrophe because DNA that was damaged by olaparib entered the mitotic phase without repairing the damaged-DNA due to the suppression of *ATM* and DNA-PKcs via *PSPC1* inhibition. Mitotic catastrophe is a phenomenon that causes cell lethality via the abnormal splitting of damaged and under-replicated DNA into daughter cells [41].

Previous studies have reported that such mechanisms were responsible for enhanced PARP inhibitor sensitivity. For example, one study showed that *ATR* inhibitor in combination with olaparib promoted greater activity than did olaparib monotherapy via mitotic catastrophe-induced cell death in *BRCA2*-mutated ovarian cancer [20]. Moreover, an ongoing phase II trial is testing the combination of olaparib and cediranib or AZD6738 (*ATR* inhibitor) for the treatment of advanced or metastatic germline BRCA-mutated breast cancer (NCT04090567). Recently, one promising DDR target, the DNA polymerase theta (*POLQ*) inhibitor, entered a phase I/II trial to treat metastatic breast cancer as a monotherapy or in combination with talazoparib (NCT04991480). In another preclinical study, olaparib also showed enhanced activity when combined with BET bromodomain inhibition via mitotic catastrophe in BRCA-proficient ovarian cancer [42]. Although targeting several genes in combination with a PARP inhibitor showed synergistic anticancer activity, to the best of our knowledge, this is the first report to show that *PSPC1* inhibition in combination with olaparib enhanced PARP inhibitor efficacy by inhibiting the DDR pathway.

Aside from targeting the DDR pathway, other strategies have also been reported to enhance PARP inhibitor efficacy in a PARP inhibitor-sensitive setting. One such strategy is targeting HR-promoting pathways, in other words, the induction of HRDness with targeted agents, such as PI3K/AKT/mTOR inhibitors in combination with PARP inhibitors [43]. To date, one phase I clinical trial of pan-class I PI3K inhibitor (buparlisib) in combination with olaparib in high-grade serous ovarian or triple-negative breast cancer has been completed (NCT01623349). BET inhibition was also reported to promote the HRD phenotype, indicating enhanced PARP inhibitor sensitivity regardless of *BRCA1/2* or RAS/RAF status [44]. A phase Ib/2 clinical trial that combined BET inhibitor ZEN-3694 and talazoparib showed a response rate of 22% in patients with triple-negative breast cancer without germline *BRCA1/2* mutations [45]. Preclinical and clinical studies have shown that PARP inhibitors exhibit synergistic effects when combined with immune checkpoint inhibitors. Several ongoing clinical trials are currently evaluating such a combination [23,46]. In addition, the targeting of RAS/RAF/MEK, c-MET, and EGFR/HER2 with their respective inhibitors is under clinical trials. A phase I clinical trial is evaluating the combination of olaparib and selumetinib (MEK inhibitor) in solid tumors with RAS pathway alterations [47]. Similarly, the efficacy of combining PARP inhibitors and c-MET tyrosine kinase inhibitors, including crizotinib (NCT04693468) and cabozantinib (NCT03425201, NCT05038839), is currently under clinical investigation.

To speculate on the potential toxicities of *PSPC1* inhibition, we attempted to search for publications regarding such outcomes in *PSPC1*-knockout mice. However, no such reports had been identified. Therefore, it is unclear how *PSPC1* inhibition affects embryonic development and normal adult tissues. Nonetheless, we did not observe any weight loss in our in vivo study, indicating that *PSPC1* inhibition had no generalized toxicities in our mouse model. Additionally, common toxicities of PARP inhibitors include nausea, vomiting, diarrhea, anemia, neutropenia, and the development of hematologic malignancies [48]. Further studies are needed to better understand toxic effects of *PSPC1* inhibition, including any overlapped toxicities with PARP inhibitors.

In conclusion, the current study found that *PSPC1* inhibition in combination with a PARP inhibitor enhanced anticancer activity in BRCA-mutated breast or ovarian cancer by regulating the DDR pathway, suggesting that *PSPC1* is a novel synthetic lethal partner with a PARP inhibitor in BRCA-mutated cancer. To date, there are no *PSPC1* inhibiting drugs available. Therefore, the development of a *PSPC1* inhibitor is a rational approach to enhance PARP inhibitor activity.

## 4. Materials and Methods

### 4.1. Drugs

Olaparib was purchased from ChemScene (Monmouth, NJ, USA) and dissolved in dimethyl sulfoxide (DMSO) (Sigma-Aldrich, St. Louis, MO, USA).

### 4.2. Cell Culture

SNU−251, an ovarian cancer cell line, was purchased from the Korean Cell Line Bank (Seoul, Korea). The breast cancer cell lines BT−474 and MDA-MB−436 were bought from American Type Culture Collection (Manassas, VA, USA). The PEO1 cell line was purchased from European Collection of Authenticated cell cultures (Porton Down, United Kingdom). All cell lines were cultured in RPMI 1640 medium (Wellgene Inc., Daegu, Korea) containing 10% heat-inactivated fetal bovine serum (Wellgene Inc., Daegu, Korea) and 1% penicillin/streptomycin solution (Wellgene Inc., Daegu, Korea), whereas PEO1 cells were supplemented with 2 mM sodium pyruvate (Gibco, Life Technologies Corporation, grand Island, NY, USA). All cells were maintained in a humidified atmosphere containing 5% CO_2_ at 37 °C.

### 4.3. Cell Viability Assay

Cell viability assay was determined using the thiazoyl blue tetrazolium bromide (MTT; Sigma, St. Louis, MO, USA) colorimetric assay. Briefly, 1500–1800 cells were seeded into a 96-well plate and incubated overnight. Next, cells were treated with different concentrations of olaparib and PSPC1 siRNA (5 nM) and incubated for 72 h. Thereafter, MTT solution (10%) was added, and the sample was incubated in the dark for 4 h at 37 °C. After incubation, the medium was removed and DMSO was added followed by incubation at room temperature (RT) for 30 min to dissolve the formazan. Finally, the absorbance was measured at 540 nm using a microplate reader (Multiskan GO Microplate Spectrophotometer). CompuSyn (ComboSyn, Inc., Paramus, NJ, USA) software was used to evaluate the half-maximal inhibitory concentration (IC_50_) and combination index (CI) values.

### 4.4. Western Blot Analysis

Cells were harvested and lysed in radioimmunoprecipitation assay (RIPA) buffer supplemented with a protease inhibitor (Roche, Basel, Switzerland) and phosphatase inhibitor cocktail (Thermo Scientific, Rockford, IL 61101, USA) for 1 h at 4 °C. For protein extraction from tumor tissues, tumors were cut into small pieces and homogenized using a homogenizer, after which RIPA buffer was added. After 1 h of incubation, the samples were centrifuged and the supernatants collected. Whole protein lysate was separated using sodium dodecyl sulfate–polyacrylamide gel electrophoresis (SDS-PAGE) and transferred onto polyvinylidene difluoride (PVDF) membranes (Millipore Burlington, MA, USA). Next, the membranes were blocked with 5% skim milk in TBS/0.1% Tween 20 for 1 h at room temperature. The membranes were then incubated with primary antibody overnight at 4 °C followed by horseradish peroxidase (HRP)-conjugated secondary antibody for 1 h. The bands were detected using an ECL Western blotting substrate (Thermo Scientific, Waltham, MA USA). A list of primary and secondary antibodies is provided in Table S2.

### 4.5. Apoptosis Assay

The apoptosis assay was assessed via flow cytometry. Briefly, cells were detached through trypsinization and washed twice with PBS. Thereafter, cells were resuspended in annexin V binding buffer (BioLegend, San Diego, CA, USA), at a concentration of 1 × 10^6^ cells/mL. Next, 100 µL of the cell suspension was transferred into a micro-centrifuge tube and stained with 5 µL of APC annexin V (BioLegend, San Diego, CA, USA) and 10 µL of propidium iodide (PI) (Invitrogen, Waltham, MA USA). After 15 min of incubation at room temperature in the dark, 400 µL of annexin V binding buffer was added to each tube. Finally, apoptotic cells were analyzed via flow cytometry (Beckman Coulter CytoFLEX, Indianapolis, USA).

### 4.6. Immunocytochemistry (ICC)

Cells were washed with PBS and fixed with 4% paraformaldehyde for 15 min on ice. After washing 3 times with PBS, permeabilization buffer (contains 0.2% triton-X in PBS) was added and incubated for 10 min at RT. Cells were then blocked with blocking buffer containing 5% BSA and 0.3% triton-X in PBS for 20 min at RT. Next, cells were incubated with the primary antibody for 2 h at RT followed by washing three times with PBS. Fluorescently labeled secondary antibody (Alexa Fluor 488 conjugated) was added and incubated for 30 min at RT in the dark. Lastly, the cells were mounted with the VECTASHIELD mounting medium containing 4′,6-diamidino-2-phenylindole [DAPI, (Vector Laboratories, Inc. Burlingame, CA, USA)] after washing with PBS. Images were then obtained using the Zeiss 510 fluorescence microscope (Zeiss, Oberkochen, Germany).

### 4.7. Cell Cycle Analysis

Cell cycle analysis was conducted using flow cytometry. In detail, cells were seeded into 60 mm culture dishes and incubated overnight. The cells were treated with olaparib (half IC_50_ or IC_50_ concentration), PSPC1 siRNA, and their combination for 72 h. After harvesting, the cells were washed twice with ice-cold PBS and fixed with 70% ethanol at −20 °C for at least 1 h or overnight. After fixation, samples were washed twice with PBS and resuspended in 300 µL of PBS containing 100 µg/mL of RNase (Signa-Aldrich, St. Louis, MO, USA) and 50 µg/mL of PI (Invitrogen, Carlsbad, CA, USA). Next, the cells were incubated in the dark for 20 min at RT. Finally, the cells were analyzed using flow cytometry (Beckman Coulter CytoFLEX, Indianapolis, IN, USA).

### 4.8. siRNA Transfection

Small interfering RNA (siRNA) transfections were performed using Lipofectamine RNAiMAX Reagent (cat# 13778-030, Invitrogen) according to the manufacturer’s protocol. Briefly, cells were seeded into a 60 mm culture dish at around 60–80% confluency at the time of transfection and incubated overnight. Next, Lipofectamine RNAiMAX Reagent and siRNA were diluted in Opti-MEM^®^ Reduced-Serum Medium (cat#31985070, ThermoFisher Scientific) separately. Thereafter, diluted siRNA was added to the diluted Lipofectamine RNAiMAX Reagent and incubated for 10–15 min at RT. Finally, the siRNA–lipid complex was added to the cells and incubated for 48 to 72 h at 37 °C in a humidified CO_2_ incubator. Lastly, we harvested the cells and performed Western blot analysis to evaluate the transfection efficiency of the siRNA.

### 4.9. Animal Studies

BALB/c nude mice were purchased from the Ja Bio Inc. (Suwon-si, Gyeonggi-do, Republic of Korea) and kept in a specific pathogen-free animal facility at CHA University (Seongnam, Korea) for at least 1 week for acclimatization before experimentation. All animals were allowed unlimited food and water and were housed under a consistent temperature of 24 ± 3 °C with a light/dark cycle of 12 h. For tumor induction, BT474 cells (1 × 10^7^ cells/mouse in 0.05 mL of PBS) were mixed with 0.05 mL of Matrigel (Corning, NY, USA) and injected subcutaneously into the mammary fat pad. After inoculation, mice were injected with estrogen valerate (3 μg/mouse) once a week. After tumor formation (approximately 75–85 mm^3^), the mice were randomly divided into four groups, namely the control siRNA (group 1), olaparib (group 2), hPSPC1-siRNA (group 3), and hPSPC1-siRNA with olaparib (group 4). Group 1 was treated with control siRNA, whereas group 2 was treated with olaparib (50 mg/kg, orally) twice a day for 16 days. Group 3 was injected with hPSPC1-siRNA (Ambion^®^ In Vivo siRNA, ThermoFisher Scientific, Waltham, MA USA) intratumorally at a concentration of 5 µg/mouse (every 3 days for 6 times), whereas group 4 was treated with olaparib (50 mg/kg) and hPSPC1-siRNA (5 µg/mouse). siRNAs were prepared using in vivo-jetPEI (cat# 201-10G, Polyplus) transfection reagent according to manufacturer’s protocol (N/P ratio 6). Tumor sizes were measured three times a week with the help of a vernier caliper, while the tumor volume was measured using the formula tumor length × tumor width × 0.5. Finally, all mice were sacrificed, and tumors were preserved for further analysis.

### 4.10. Genomics of Drug Sensitivity in Cancer (GDSC) Analysis

Gene expression and drug screening data for the cell lines were downloaded from the GDSC website [49]. A total of 19 ovarian cancer cell lines were used to analyze the association between PSPC1 mRNA levels and olaparib sensitivity, which was defined as IC_50_ ≤ 3.2 µM. A list of the 19 ovarian cancer cell lines used herein is presented in Table S3.

### 4.11. Public Gene Expression Profiling Data Sets in Breast and Ovarian Cancer

To determine whether PSPC1 mRNA expression levels were associated with prognosis in patients with breast and ovarian cancer, we analyzed two independent public mRNA expression data sets of curatively resected breast cancer (GSE17907 and GSE11121) and three independent public mRNA expression data sets of primary debulked ovarian cancer (GSE26712, TCGA, and GSE9891) using the online platform Kaplan–Meier plotter [50]: https://kmplot.com/analysis/ (accessed on 01 August 2023). Affymetrix Genechip Human Genome U133 Arrays were used for all these data sets. Affymetrix ID 218371 was selected for PSPC1. PSPC1 mRNA expression levels were divided into two groups, “PSPC1-High” and “PSPC1-Low” with the median as the cutoff. Recurrence-free survival (RFS) or overall survival (OS) were analyzed based on PSPC1 mRNA expression level.

### 4.12. Statistical Analysis

Student’s *t*-test analysis was performed using SPSS version 20 (IBM SPSS Statistics 20, Armonk, NY, USA). Student’s *t*-test was performed to compare the two groups in the gene expression levels according to olaparib sensitivity using the GDSC database. *p*-values < 0.05 were considered statistically significant. One-way analysis of variance (ANOVA) followed by Tukey’s HSD test was performed using GraphPad Prism (version 8.0.2, Boston, MA). One-way ANOVA was used to compare multiple groups in the apoptosis assay, ICC, and in vivo tumor growth inhibition assay. Statistical significance was defined as * *p* < 0.05, ** *p* < 0.01, *** *p* < 0.001 and **** *p* < 0.0001. *p*-values < 0.05 were considered statistically significant. RFS was defined as the time from curative surgery to cancer recurrence or the last date at which the patient was known to be free of recurrence (censoring time). OS was defined as the time from diagnosis to death or the last date at which the patient was known to be alive (censoring time). RFS and OS were calculated using the Kaplan–Meier method. A log rank test was used to compare RFS and OS between groups. All *p*-values were two sided and *p*-values < 0.05 were considered statistically significant.

## Data Availability

The original data have been included in the paper and can be obtained from the corresponding author upon reasonable inquiry.

## Supplementary Materials

The following supporting information can be downloaded at: https://www.mdpi.com/article/10.3390/ijms242317086/s1.
