# Supplementary material for: PSPC1 Inhibition Synergizes with Poly(ADP-ribose) Polymerase Inhibitors in a Preclinical Model of BRCA-Mutated Breast/Ovarian Cancer
Source: Int J Mol Sci. 2023 Dec 3;24(23):17086. doi: 10.3390/ijms242317086 (PMC10707354; doi:10.3390/ijms242317086)
Supplement: Supplementary file 1 [file ijms-24-17086-s001.zip › ijms-2749470-supplementary/Supplementary figure and tables.pdf]

International Journal of *Molecular Sciences*

MDPI

Article

PSPC1 Inhibition Synergizes with Poly (ADP-ribose) Polymerase Inhibitors in a Preclinical Model of BRCA-mutated Breast/Ovarian Cancer

Mithun Ghosh [1], Min Sil Kang[1], Nar Bahadur Katuwal[1], Sa Deok Hong[1], Yeong Gyu Jeong[1], Seong Min Park[1], Seul-Gi Kim[2], Yong Wha Moon[2,*]

[1] Department of Biomedical Science, The Graduate School, CHA University, Seongnam-si, Republic of Korea (13488); mithunghoshmg@gmail.com (M.G.); rkdalstlf1097@gmail.com (M.S.K.); narbahadurkatwal@gmail.com (N.B.K); duggy126@gmail.com (S.D.H.); younggyu6337@gmail.com (Y.G.J.); hoy105@naver.com (S.M.P.)

2 Hematology and Oncology, Department of Internal Medicine, CHA Bundang Medical Center, CHA University, Seongnam-si, Republic of Korea (13496); sophia311@chamc.co.kr (S.G.K.)

* Correspondence: ymoon@cha.ac.kr (Y.W.M.); Tel: +82-31-780-3436 (Y.W.M), Fax: +82-31-780-3929 (Y.W.M)

Citation: To be added by editorial staff during production.

Academic Editor: Firstname Lastname

Received: date
Revised: date
Accepted: date
Published: date

Copyright: © 2023 by the authors. Submitted for possible open access publication under the terms and conditions of the Creative Commons Attribution (CC BY) license (https://creativecommons.org/licenses/by/4.0/).

Supplementary figure

BT-474

(A)

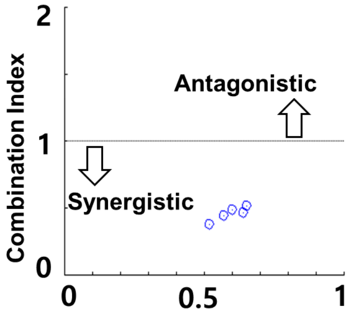

Olaparib (μM)	PSPC1 siRNA (μM)	Effect	CI
30	0.005	0.521	0.381
50	0.005	0.570	0.442
70	0.005	0.601	0.492
90	0.005	0.641	0.466
110	0.005	0.653	0.516

*CI >1: Antagonism, CI =1: Additive, CI <1: Synergism

SNU-251

(B)

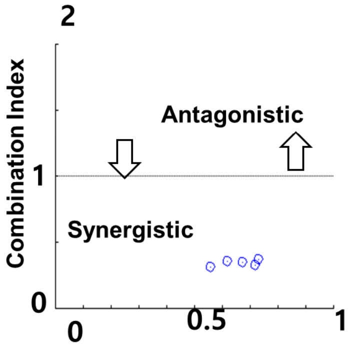

Olaparib (μM)	PSPC1 siRNA (μM)	Effect	CI
30	0.005	0.557	0.317
50	0.005	0.619	0.360
70	0.005	0.674	0.353
90	0.005	0.719	0.331
110	0.005	0.730	0.371

*CI >1: Antagonism, CI =1: Additive, CI <1: Synergism

Supplementary figure S1. (A) CI value of MTT assay in BT-474 cells. (B) CI value of MTT assay in SNU-251 cells

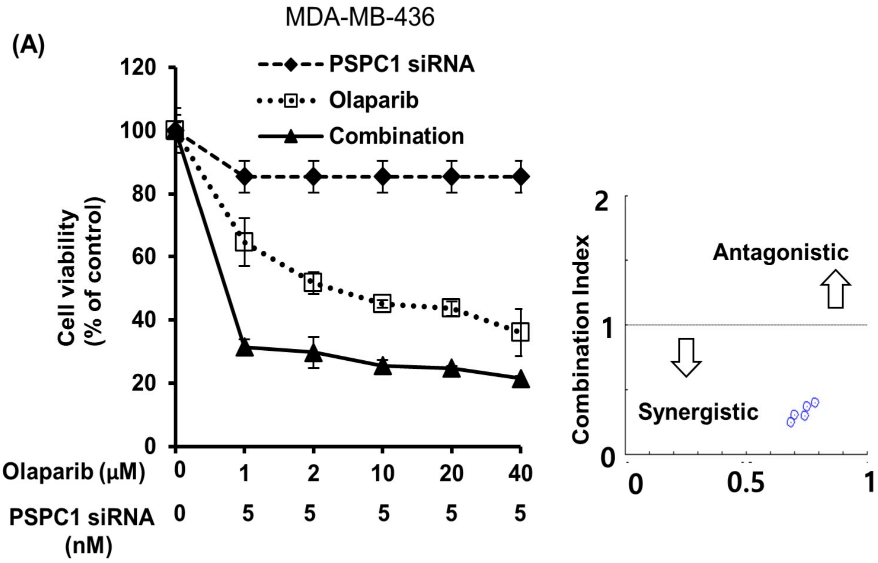

Olaparib (μM)	PSPC1 siRNA (μM)	Effect	CI
1	0.005	0.686	0.249
5	0.005	0.703	0.306
10	0.005	0.744	0.297
20	0.005	0.752	0.374
40	0.005	0.785	0.400

*CI >1: Antagonism, CI =1: Additive, CI <1: Synergism

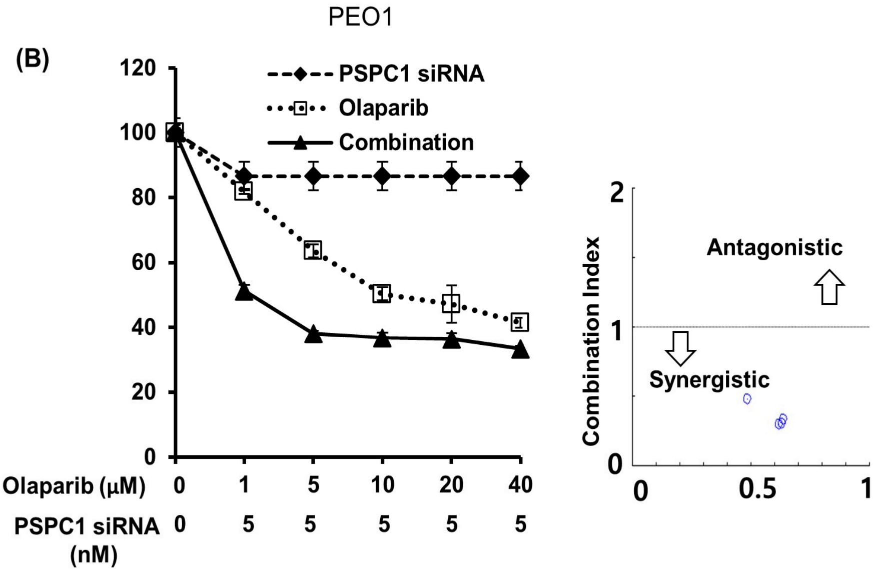

Olaparib (μM)	PSPC1 siRNA (μM)	Effect	CI
1	0.005	0.487	0.483
5	0.005	0.619	0.299
10	0.005	0.633	0.304
20	0.005	0.637	0.340
40	0.005	0.667	0.317

*CI >1: Antagonism, CI =1: Additive, CI <1: Synergism

Supplementary figure S2. (A-B) MTT assay showed that combined PSPC1 siRNA and olaparib enhanced the antiproliferative effects in (A) MDA-MB-436 and (B) PEO1 cells. Cells were treated with 5 nM of PSPC1 siRNA and indicated concentrations of olaparib and incubated for 72 hours. Middle and right panel exhibited combination index (CI) and CI values respectively.

Supplementary Tables

Supplementary table S1: Information about histology, subtype, and BRCA1/2 mutation of BT-474 and SNU-251 cell.

Cell	BT-474	MDA-MB-436	SNU-251	PEO1
Cancer type	Breast	Breast	Ovarian	Ovarian
Histology	IDC (Invasive ductal carcinoma)	Invasive breast carcinoma	Endometroid carcinoma	Cystadenocarcinoma
Subtype	ER+HER2+	Triple negative		
BRCA mutation	BRCA2-mutated	BRCA1-mutated	BRCA1-mutated	BRCA2-mutated
	9281C > A; (S3094*)	c.5277 + 1G>A	Codon 1815 nonsense mutation, TGG(Trp) →TGA (stop)	p.Tyr1655Ter (c.4965C>G)
	Pathogenic	Pathogenic	Pathogenic	Pathogenic

Supplementary table S2: List of Primary and secondary antibodies were used in western blot and immunocytochemistry.

Antibody	Host species	Dilution	Company (catalog#)	Application
PSPC1	Mouse	1:100	sc-374181, Santa Cruz	WB
Caspase-3	Rabbit	1:1000	9662, Cell signaling	WB
DNA-PKcs	Rabbit	1:1000	ab32566, Abcam	WB
ATM	Mouse	1:200	sc-23921, Santa Cruz	WB
p-ATM	Mouse	1:200	sc-47739, Santa Cruz	WB
ɣH2AX	Rabbit	1:1000	5437, Cell signaling	WB, ICC
p-cdc25C	Rabbit	1:1000	4901, Cell signaling	WB
p-cdc2	Rabbit	1:1000	4539, Cell signaling	WB
GAPDH	Rabbit	1:1000	2118, Cell signaling	WB
Anti-Rabbit HRP	Goat	1:5000	GTX213110-01, Gene Tex	WB
Anti-Mouse HRP	Goat	1:5000	GTX213111-01, Gene Tex	WB

Supplementary table S3: A list of 19 BRCA1/2-mutated ovarian cancer cell lines in the GDSC database and olaparib IC_{50}. BRCA1/2 mutation was determined from COSMIC website (https://cancer.sanger.ac.uk)

Cell line	Cosmic ID	Cancer type	BRCA1/2 mutation	Olaparib IC_{50} (nmol)
SK-OV-3	905959	Ovary	BRCA2	4750.87
IGROV-1	905968	Ovary	BRCA1	4841.77
OVCAR-4	905990	Ovary	BRCA2	4265.86
EFO-27	906852	Ovary	BRCA2	3511.01
OC-314	909257	Ovary	BRCA1/2	3676.41
SW626	909753	Ovary	BRCA2	4543.61
KURAMOCH	909975	Ovary	BRCA2	4623.21
OAW-42	910548	Ovary	BRCA2	3413.52
KGN	924186	Ovary	BRCA1	4553.62
OVMIU	1240200	Ovary	BRCA2	4663.57
TOV-21G	1240222	Ovary	BRCA2	846.66
RKN	1298539	Ovary	BRCA2	3681.74
JHOS-2	1479995	Ovary	BRCA1	4094.88
JHOS-3	1480358	Ovary	BRCA1	4997.17
JHOS-4	1480359	Ovary	BRCA1	5189.69
OV-17R	1480361	Ovary	BRCA2	4904.96
OVCA420	1480364	Ovary	BRCA1	5025.65
PEO1	1480372	Ovary	BRCA2	4243.23
UWB1.289	1480374	Ovary	BRCA1	4009.04
