# Supplementary figures and images for: PSPC1 Inhibition Synergizes with Poly(ADP-ribose) Polymerase Inhibitors in a Preclinical Model of BRCA-Mutated Breast/Ovarian Cancer
Source: Int J Mol Sci. 2023 Dec 3;24(23):17086. doi: 10.3390/ijms242317086 (PMC10707354; doi:10.3390/ijms242317086)

Figure 1

(F)

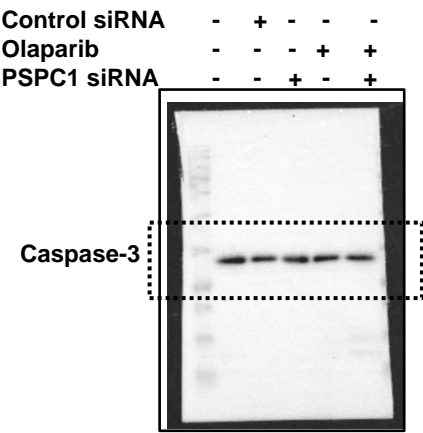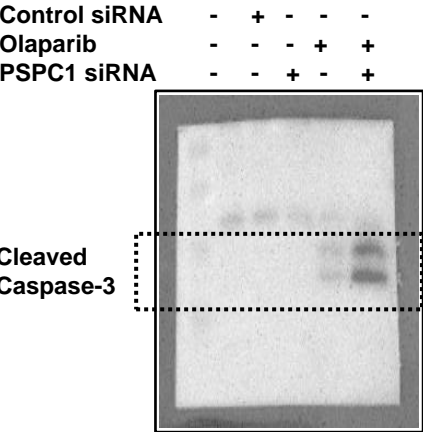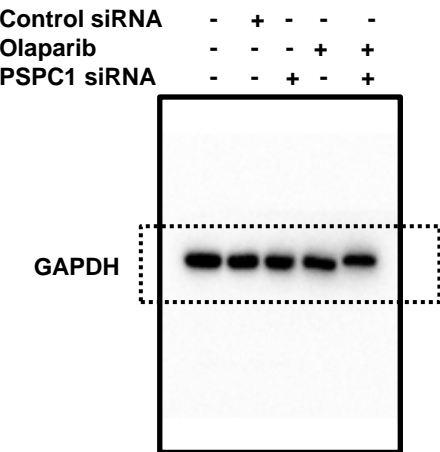

(G)

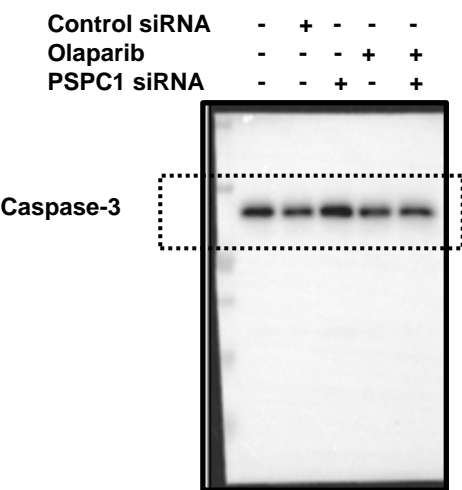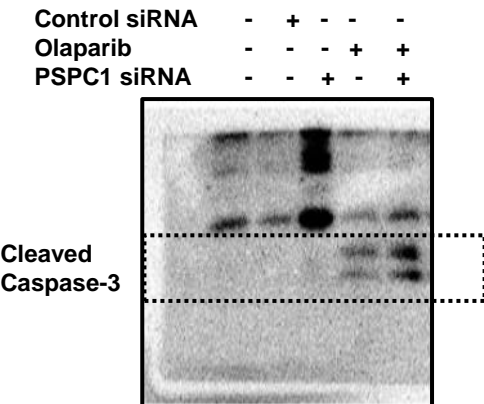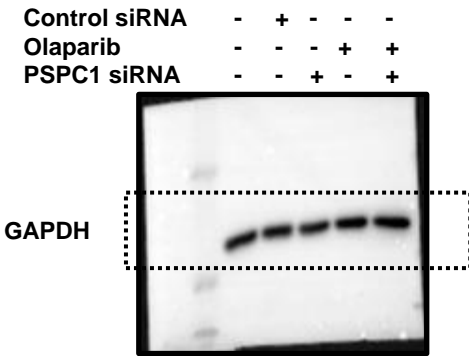

**Figure 2**

**(A)**

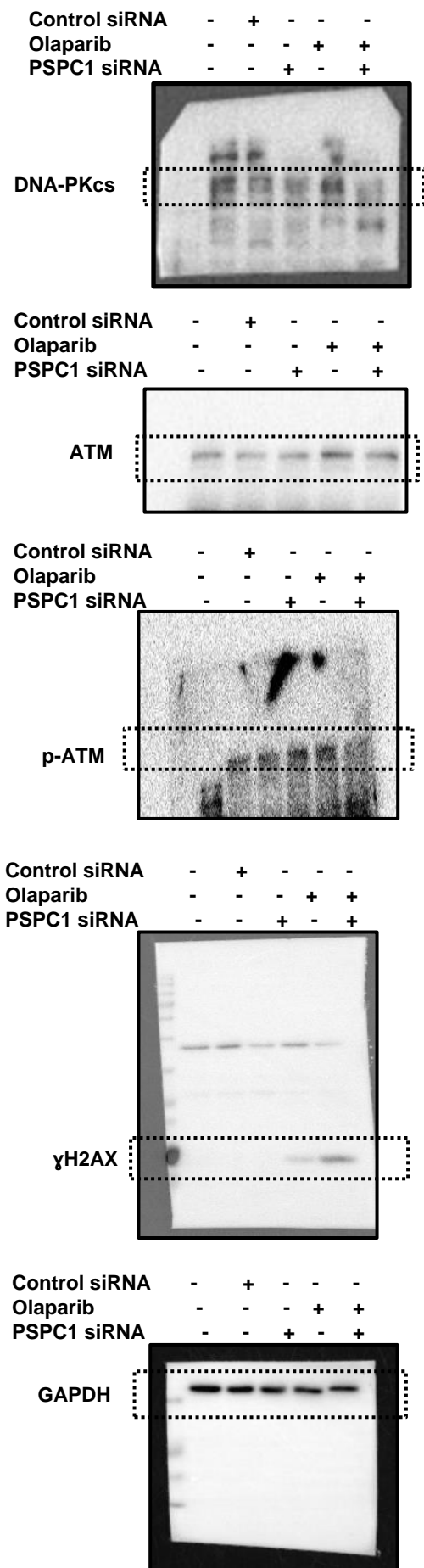

**(B)**

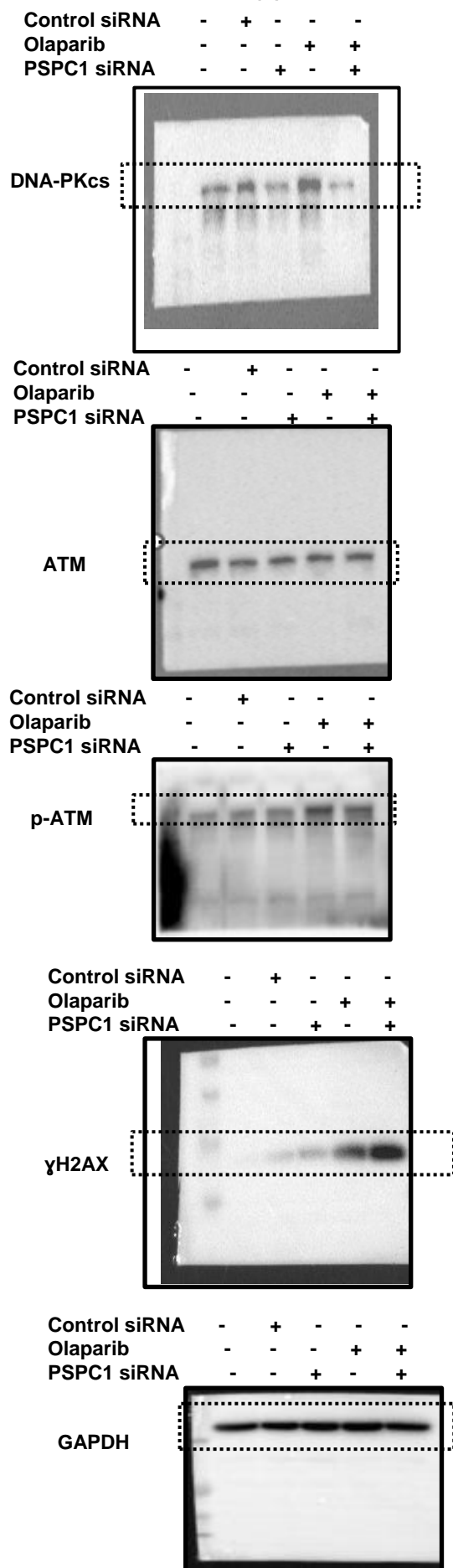

Figure 3

(C)

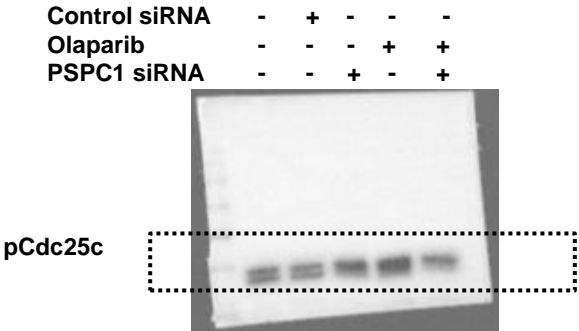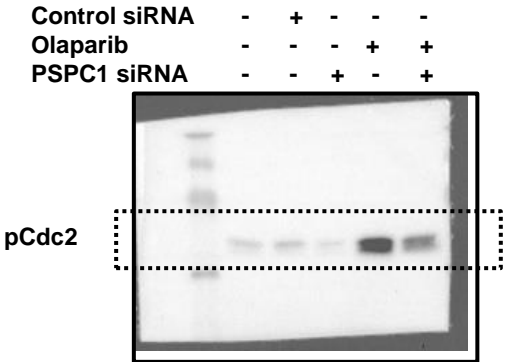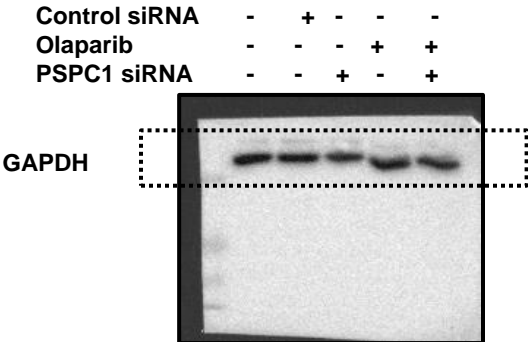

(D)

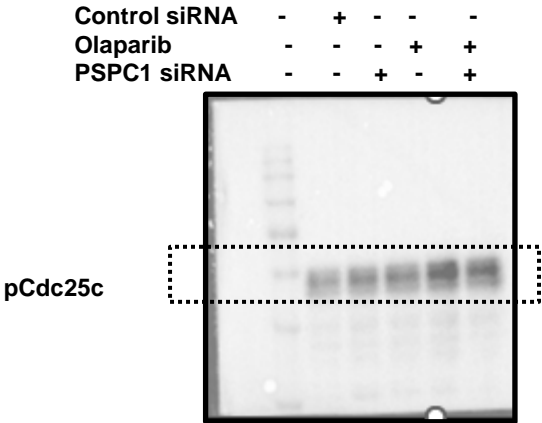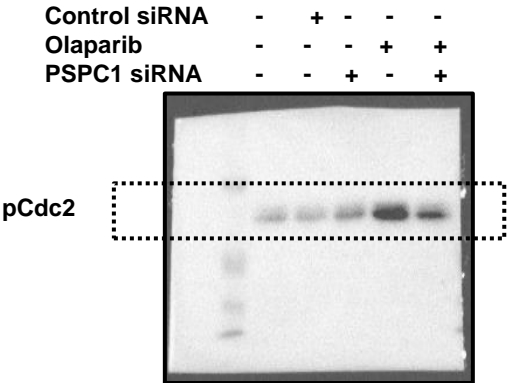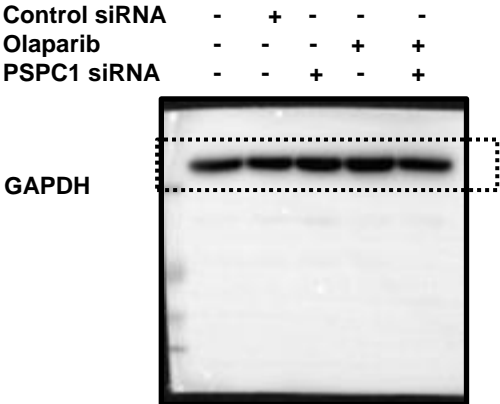

**Figure 4**

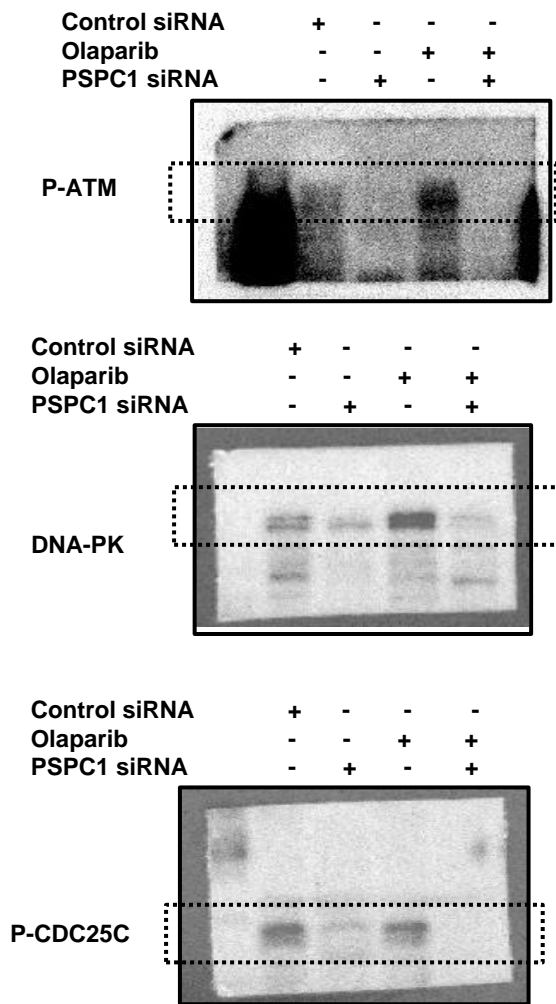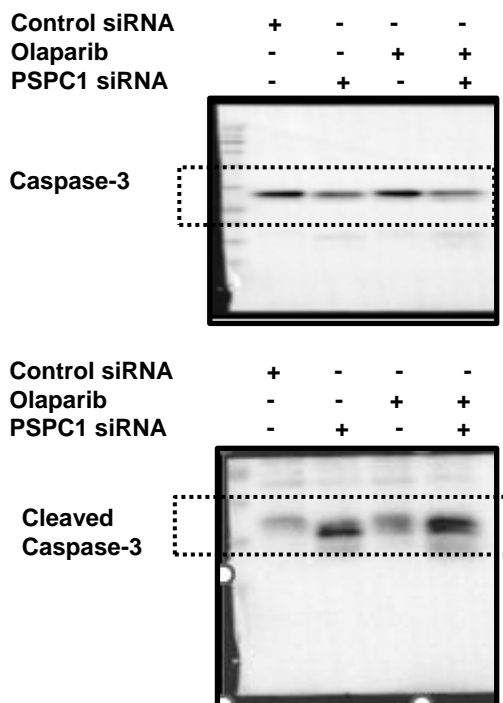

**(F)**

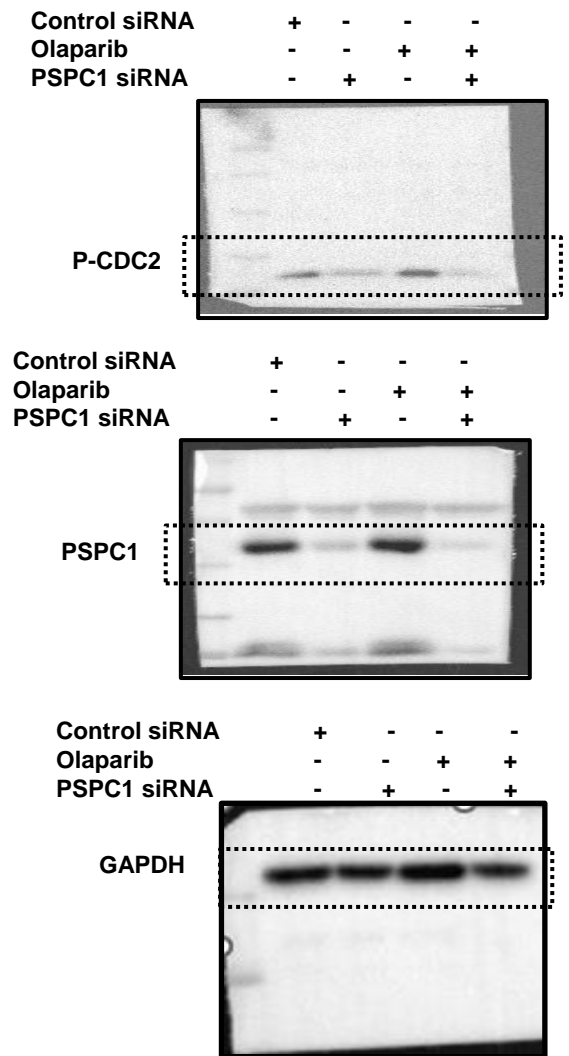

**(G)**

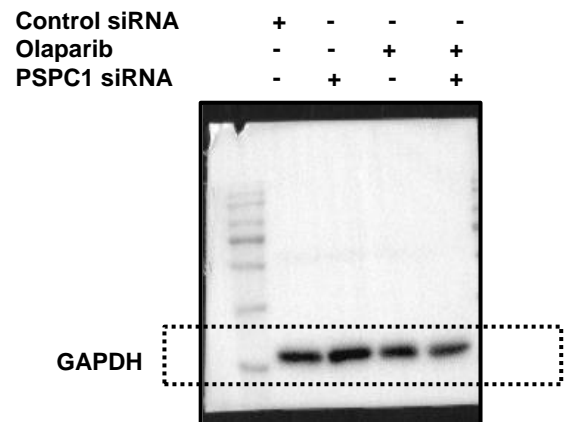

Supplement: Supplementary file 1 [file ijms-24-17086-s001.zip › ijms-2749470-supplementary/Uncropped imunoblot.pdf]
